# Significance of serum NLRP3 as a potential predictor of 5-year death in hemodialysis patients: A prospective observational cohort study

**DOI:** 10.1097/MD.0000000000039185

**Published:** 2024-08-02

**Authors:** Yi Jiang, Yandan Xu, Qiuli Wang, Zhiwei Chen, Chunya Liu

**Affiliations:** a Department of Nephrology, The Quzhou Affiliated Hospital of Wenzhou Medical University, Quzhou People’s Hospital, Quzhou, China; b Department of Nephrology, Quzhou KeCheng People’s Hospital, Quzhou, China; c Traditional Chinese Medicine Department, Quzhou Hospital of Zhejiang Medical and Health Group, Quzhou, China.

**Keywords:** biomarkers, hemodialysis, mortality, NLRP3, overall survival

## Abstract

Nucleotide-binding oligomerization domain-like receptor family pyrin domain-containing 3 (NLRP3) is involved in inflammatory response. This study was done to explore the role of serum NLRP3 as a predictive biomarker of death after hemodialysis. In this prospective observational study of 331 patients undergoing maintenance hemodialysis, serum NLRP3 levels were measured. Univariate analysis and multivariate analysis were sequentially performed to determine predictors of 5-year death after hemodialysis. Age, major adverse cardiac and cerebral events (MACCE), and serum NLRP3 levels independently predicted 5-year mortality and overall survival (all *P* < .05). No interactions were found between serum NLRP3 levels and other variables, such as age, gender, hypertension, diabetes mellitus, primary renal diseases, and MACCE (all *P* interaction > .05). Serum NLRP3 levels were linearly correlated with risk of death and overall survival under restricted cubic spline (both *P* > .05) and substantially discriminated patients at risk of death under receiver operating characteristic curve (*P* < .001). Two models, in which age, MACCE, and serum NLRP3 were combined, were built to predict 5-year mortality and overall survival. The mortality prediction model had significantly higher predictive ability than age, AMCCE, and serum NLRP3 alone under receiver operating characteristic curve (all *P* < .05). The models, which were graphically represented by nomograms, performed well under calibration curve and decision curve. Serum NLRP3 levels are independently related to 5-year mortality and overall survival of patients after hemodialysis, suggesting that serum NLRP3 may be a potential prognostic biomarker of hemodialysis patients.

## 1. Introduction

Chronic kidney disease (CKD) is a major public health challenge worldwide.^[1]^ CKD easily progresses into end-stage renal disease (ESRD), which refers to the inability of the kidneys to maintain fluid, electrolyte, and waste balance in the body.^[2]^ ESRD greatly contributes to death of CKD patients.^[3,4]^ Hemodialysis is widely recognized as an effective and frequently employed renal replacement therapy for ESRD patients.^[5]^ ESRD patients in need of hemodialysis very frequently suffer from multiorgan injuries, which are related to systemic inflammation, oxidative stress, cellular apoptosis, etc.^[6]^ Those patients stay at a high risk of midterm death, with 3-year mortality ranging from 32% to 45%.^[7–11]^ Therefore, prognosis prediction is a very important step in clinical work of ESRD. A growing body of researchers has paid attention to biomarkers with respect to their prognostic predictive ability in hemodialysis patients.^[12–14]^

The nucleotide-binding oligomerization domain-like receptor family pyrin domain-containing 3 (NLRP3) inflammasome is a critical component of the innate immune system and participates in inflammatory injury by mediating caspase-1 activation and regulating the release of pro-inflammatory cytokines interleukin-1β and interleukin-18.^[15,16]^ NLRP3 can be released from injured tissues under some pathological conditions, such as acute myocardial infarction, sepsis, and aneurysmal subarachnoid hemorrhage; and elevated circulating NLRP3 levels were tightly related to disease severity and clinical outcomes of those diseases.^[17–19]^ Alternatively, activation of NLRP3 inflammasome dramatically aggravated renal injury.^[20]^ Also, NLRP3 inflammasome was activated in ESRD.^[21]^ Therefore, serum NLRP3 may be a prognostic biomarker of ESRD patients undergoing hemodialysis. Here, serum NLRP3 levels were quantified at first-time hemodialysis of ESRD patients to further investigate its predictive significance for 5-year death after hemodialysis.

## 2. Materials and methods

### 2.1. Study design, subject selection, and ethical consent

In this prospective observational study, maintenance hemodialysis patients were consecutively enrolled between January 2014 and June 2017 at the Quzhou Affiliated Hospital of Wenzhou Medical University. The exclusion criteria were as follows: dialysis duration <3 months; irregular dialysis; and presence of other specific conditions or diseases, for example, malignancies, infection within recent a month, and severe cardio-cerebral diseases. The ethical guidelines of the Declaration of Helsinki and its later amendments were strictly obeyed during the study. The study protocol was endorsed by the Institutional Review Committee at the Quzhou Affiliated Hospital of Wenzhou Medical University (approval number: LW2014-012). Participants volunteered to participate in the current study, and the legal representatives of patients in advance signed informed consent.

### 2.2. Data collections

Some baseline data were recorded, including age, gender, hypertension, and diabetes mellitus. During treatments, major adverse cardiac and cerebral events (MACCE) were identified. MACCE encompassed acute coronary syndrome, heart failure, cardiac death, and stroke.^[22,23]^ In addition, urea reduction rate, post-dialysis weight, Kt/V, normalized protein catabolic rate, and residual creatinine clearance were calculated and then registered. Primary renal diseases were divided into chronic glomerulonephritis and other types. The patients were followed up until death or the completion of 5 years after first hemodialysis. The outcome parameter was death within 5 years.

### 2.3. immune analysis

Blood of patients was drawn via the median cubital vein. Some conventional laboratory data, including blood levels of intact parathyroid hormone, creatinine, albumin, hemoglobin, alkaline phosphatase, C-reactive protein, cholesterol, triglyceride, calcium and phosphate, hematocrit, and blood white blood cell counts were measured using the routine test methods. For measurement of serum NLRP3 levels, blood samples were put in 5 mL gel-containing biochemistry tubes (Hebei Free Trade Zone Shenghong Medical Equipment Co., Ltd., China), and after centrifugation, an aliquot of serum sample was extracted and afterward preserved in Eppendorf tubes (Eppendorf Tubes® BioBased, China) at a −80°C freezer for further immune analysis. Serum NLRP3 levels were quantified in batches. Blood samples, which were thawed every 3 months, were used for measuring serum NLRP3 levels. The sandwich enzyme-linked immunosorbent assay kit was purchased from Shanghai Zhenke Biotech Co., Ltd. (Shanghai, China). Detection range of the reagent kit was from 0.1 to 10 ng/mL. Its intra-assay coefficient of variation was <15%, with inter-assay coefficient of variation of <15%. A sandwich enzyme-linked immunosorbent assay reader was used to assess 450 nm optical density (Infinite M200 pro; Tecan®, Salzburg, Austria). Using blind method, all quantifications were in duplicate and completed by the same experienced technician. Double measurements were averaged for statistical analysis.

### 2.4. Statistical analysis

The MedCalc statistical software version 17.4 (MedCalc Software, Mariakerke, Belgium), SPSS statistical package version 20.0 (SPSS Inc., Chicago, IL), and R software version 4.3.0 (https://www.r-project.org) were used for statistical analysis. All graphs were plotted using the GraphPad Prism statistical software version 8 (GraphPad Software, San Diego, CA). Data were presented as counts (percentages) if they were categorical variables, means (standard deviations) if they were normally distributed continuous variables, and medians (interquartile ranges) if they were non-normally distributed continuous variables. Statistical methods for comparing data between 2 groups included the Pearson Chi-square test, independent *t* test, and Mann–Whitney *U* test. Under the restricted cubic spline, linear correlations of serum NLRP3 levels with risks of 5-year death and overall survival were reported. The binary logistic regression model, in which 5-year mortality was selected as the dependent variable, was built to discern the factors, which independently influenced 5-year mortality of hemodialysis patients. Associations were presented as odds ratios with the corresponding 95% confidence intervals (95% CIs). Discriminatory ability was assessed under receiver operating characteristic (ROC) curve. The results were reported as area under ROC curve (AUC) and Z test was done for comparisons of AUCs. The prediction model, in which the independent factors of 5-year mortality were incorporated, was described using the nomogram. The prediction efficiency of the model was assessed using the ROC curve, its prediction fit was determined using the Hosmer–Lemeshow goodness of fit statistics and calibration curve analysis, and its clinical effectiveness was investigated using the decision curve analysis. Survival curves were generated according to the Kaplan–Meier method. The log-rank test was performed to complete the comparison of 5-year overall survival between 4 subgroups, which was formed according to median and lower-upper quartile values of serum NLRP3 levels. The multivariate Cox proportional hazard model, in which 5-year overall survival time was regarded as the dependent variable, was established to ascertain independent predictive parameters. The Cox regression coefficients were used to generate a nomogram. The calibration curve for the 5-year survival probability was used to evaluate the relationship between the nomogram prediction and actual observation. The decision curve was used to evaluate the clinical utility. Using subgroup analysis, interactions with age, gender, hypertension, diabetes mellitus, primary renal diseases, and MACCE were investigated. Using the MedCalc statistical software version 17.4 (MedCalc Software), the sample size was sufficient for clinical analysis. The 2-sided *P* values < .05 were deemed as statistically significant differences.

## 3. Results

### 3.1. Participant characteristics

**Figure 1.**
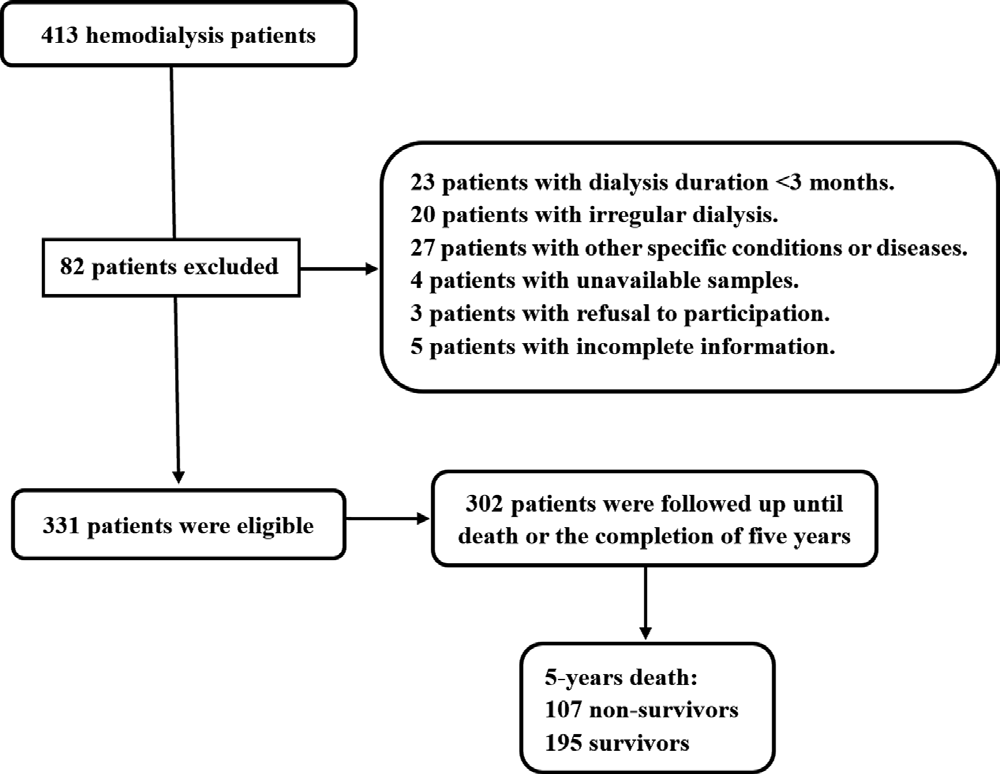
Flowing diagram for selecting appropriate patients with hemodialysis. A cohort of 413 patients underwent an initial assessment in accordance with the enrollment criteria, and 331 patients were finally selected as eligible subjects for further clinical study after 82 patients were removed from this study. Among them, 302 patients were followed up until death or the completion of 5 yr.

During the study period, an aggregate of 413 patients with hemodialysis were initially recruited, afterward, 82 patients were excluded because of reasons outlined in Figure 1, and finally, 331 patients were analyzed. Among them, 302 patients were followed up until death or the completion of 5 years. In Table 1, there were non-statistically significant differences in terms of demographic, clinical, and biochemical data between those 302 and 331 patients (all *P* > .05).

**Table 1:** Basic data of the hemodialysis patients.

Variables	The first group	*The second group	*P* values
Number	331	302	
Age (yr)	69.6 ± 7.0	69.6 ± 7.0	.997
Gender (male/female)	119/212	108/194	.960
Hypertension	183 (55.3%)	170 (56.3%)	.799
Diabetes mellitus	204 (61.6%)	185 (61.3%)	.923
Primary renal diseases			.963
Chronic glomerulonephritis	142 (42.9%)	129 (42.7%)	
Others	189 (57.1%)	173 (57.3%)	
Major adverse cardiac and cerebral events	168 (50.8%)	154 (51.0%)	.952
Laboratory data			
Blood intact parathyroid hormone levels (pg/mL)	368.9 (155.9–715.1)	349.7 (155.9–694.4)	.879
Blood creatinine levels (mg/dL)	9.2 (8.1–11.0)	9.2 (8.0–10.9)	.853
Blood albumin levels (mg/dL)	3.4 ± 0.8	3.4 ± 0.8	.908
Blood hemoglobin levels (g/dL)	10.4 ± 2.5	10.3 ± 2.4	.867
Hematocrit (%)	28.2 (24.2–33.6)	28.1 (24.2–33.6)	.852
Blood alkaline phosphatase levels (IU/L)	87.2 ± 20.8	87.0 ± 20.4	.870
Blood C-reactive protein levels (mg/dL)	1.1 (0.7–1.5)	1.0 (0.7–1.4)	.833
Blood cholesterol levels (mg/dL)	176.5 (153.7–210.7)	176.5 (151.8–210.7)	.792
Blood triglyceride levels (mg/dL)	109.8 (71.9–166.8)	109.6 (69.3–165.5)	.759
Blood leucocyte count (×10^9^/L)	7.2 ± 1.9	7.2 ± 1.9	.990
Blood calcium levels (mg/dL)	8.9 (7.7–10.6)	8.9 (7.6–10.6)	.863
Blood phosphate levels (mg/dL)	4.8 (4.1–5.7)	4.7 (4.1–5.7)	.864
Blood calcium × phosphate levels	53.0 (45.6–63.3)	44.2 (37.9–52.8)	.820
Urea reduction rate (%)	0.7 ± 0.2	0.7 ± 0.2	.890
Post-dialysis weight (kg)	53.0 (45.6–63.3)	52.4 (45.6–62.7)	.846
Kt/V (Daugirdas)	1.6 ± 0.4	1.6 ± 0.4	.996
Normalized protein catabolic rate (g/kg/d)	1.1 ± 0.3	1.1 ± 0.3	.866
Residual creatinine clearance (mL/min)	5.3 (4.6–6.3)	5.3 (4.6–6.3)	.895
Serum NLRP3 levels (ng/mL)	1.9 (1.2–3.0)	1.9 (1.2–3.0)	.833

Data were shown as counts (percentages), means ± standard deviations or medians (upper-lower quartiles) where appropriate. Intergroup comparisons were done using the Chi-square test, Fisher exact test, Student *t* test, or Mann–Whitney test as appropriate.

NLRP3 = nucleotide-binding oligomerization domain-like receptor family pyrin domain-containing 3.

* A total of 331 hemodialysis patients were eligible for final analysis (the first group) and 302 patients were followed up until death or the completion of 5 years after hemodialysis (the second group).

### 3.2. Serum NLRP3 levels and 5-year mortality of patients with hemodialysis

**Figure 2.**
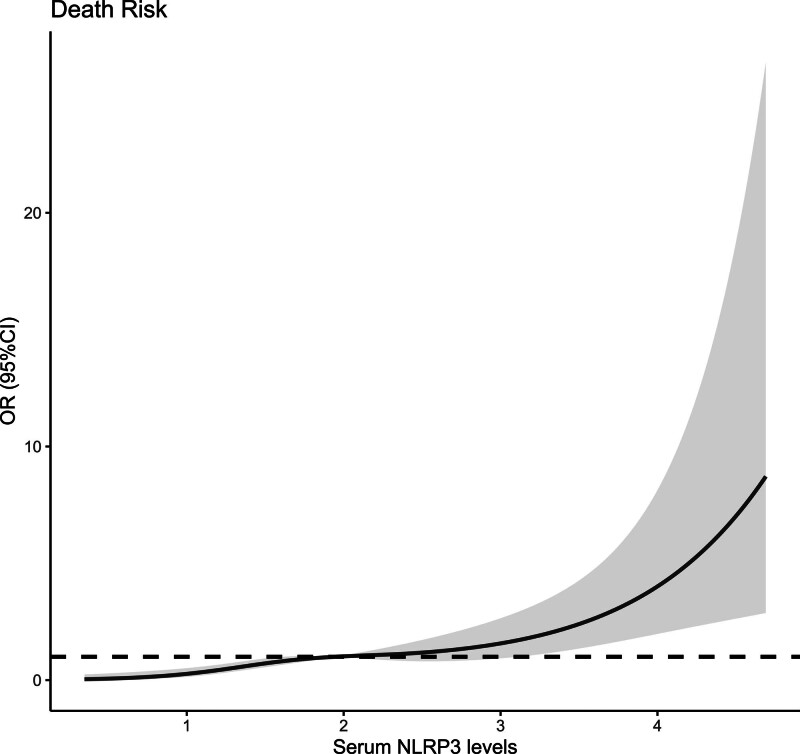
Restricted cubic spline showing the linear relationship between serum nucleotide-binding oligomerization domain-like receptor family pyrin domain-containing 3 levels and the risk of 5-yr death after hemodialysis. Serum nucleotide-binding oligomerization domain-like receptor family pyrin domain-containing 3 levels were linearly related to the risk of 5-yr death in hemodialysis patients (*P* > .05). 95% CI = 95% confidence interval, NLRP3 = nucleotide-binding oligomerization domain-like receptor family pyrin domain-containing 3, OR = odds ratio.

**Figure 3.**
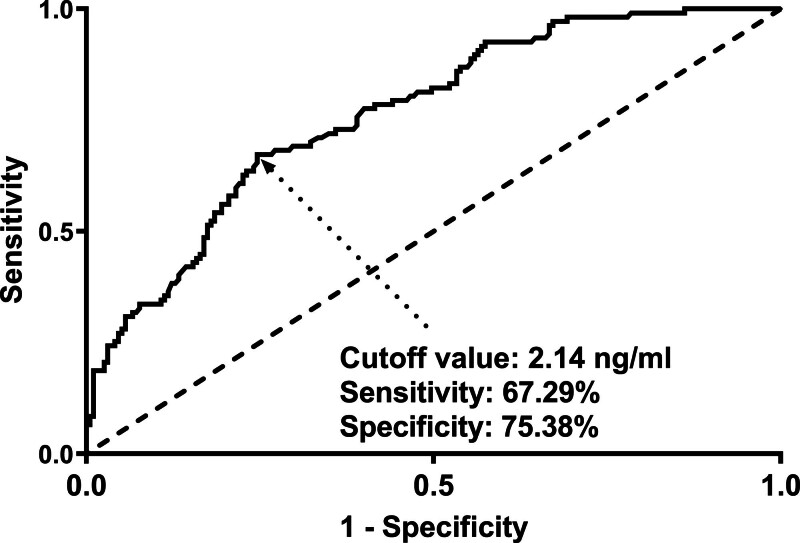
Receiver operating characteristic curve of serum nucleotide-binding oligomerization domain-like receptor family pyrin domain-containing 3 levels for predicting 5-yr mortality of hemodialysis patients. Using the Youden method, serum nucleotide-binding oligomerization domain-like receptor family pyrin domain-containing 3 levels >2.14 ng/mL distinguished the risk of death in hemodialysis patients with medium-high specificity and sensitivity.

**Figure 4.**
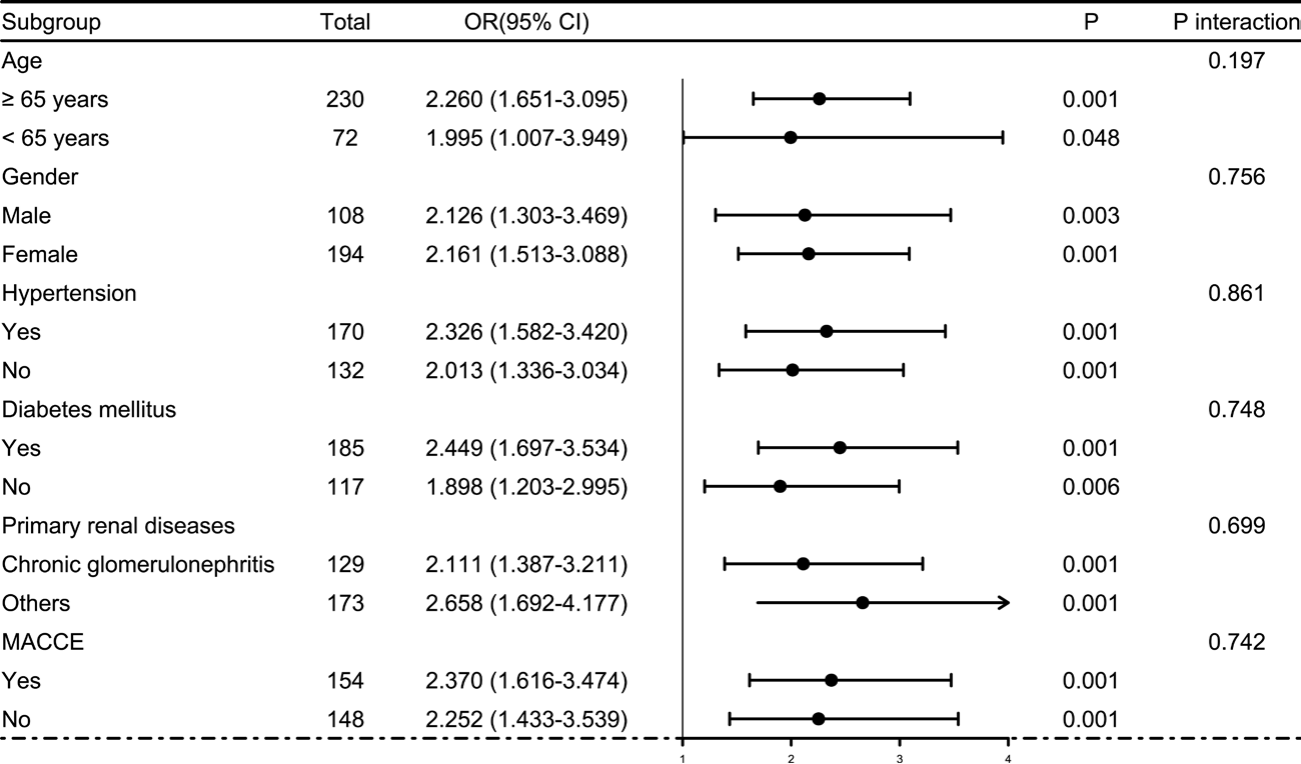
Subgroup analysis discerning interaction between serum nucleotide-binding oligomerization domain-like receptor family pyrin domain-containing 3 levels and other variables for predicting 5-yr mortality of hemodialysis patients. No significant interactions were found between serum nucleotide-binding oligomerization domain-like receptor family pyrin domain-containing 3 levels and age, gender, hypertension, diabetes mellitus, primary renal diseases, and major adverse cardiac and cerebral events (all *P* > .05). 95% CI = 95% confidence interval, MACCE = major adverse cardiac and cerebral events, OR = odds ratio.

**Figure 5.**
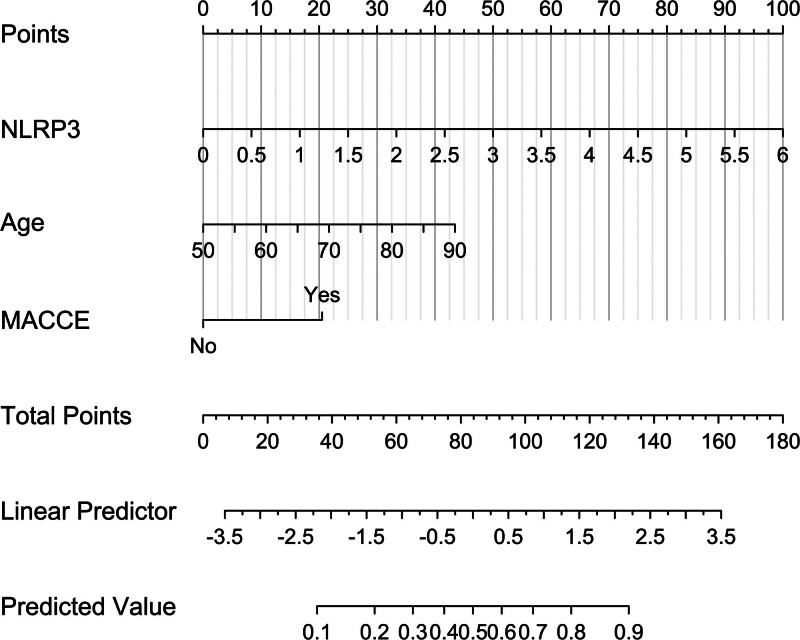
Nomogram describing the prediction model of the risk of 5-yr death in hemodialysis patients. A nomogram was constructed to visually display the prediction model of 5-yr mortality of patients with hemodialysis, in which serum nucleotide-binding oligomerization domain-like receptor family pyrin domain-containing 3 levels, age, and major adverse cardiac and cerebral events were integrated. MACCE = major adverse cardiac and cerebral events, NLRP3 = nucleotide-binding oligomerization domain-like receptor family pyrin domain-containing 3.

**Figure 6.**
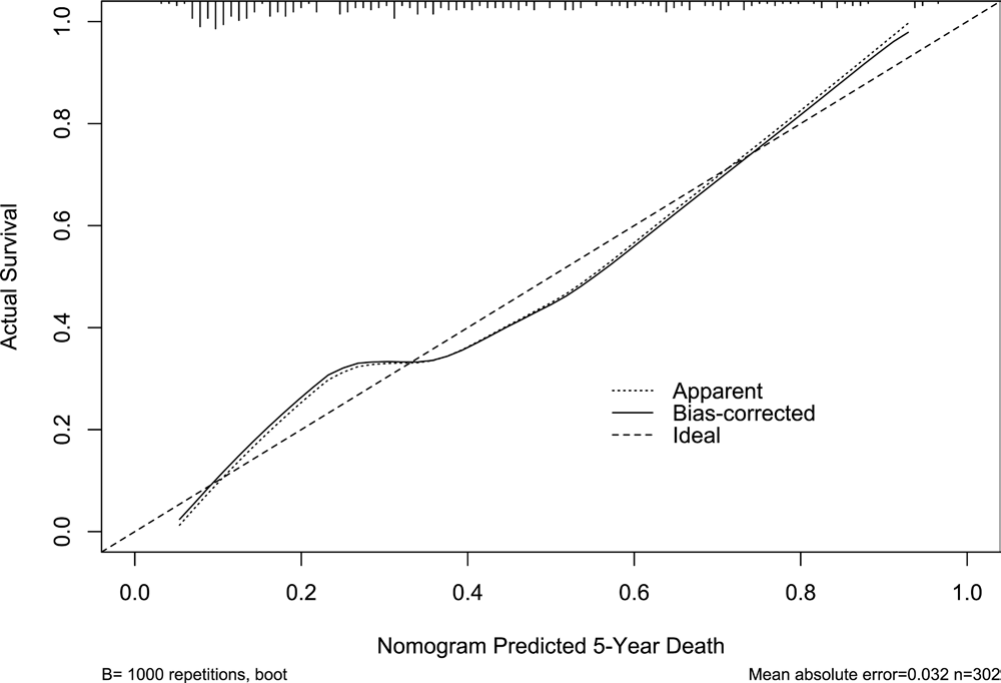
Calibration curve showing stability of the predictive model of 5-yr mortality of patients with hemodialysis. The prediction model remained stable for the prediction of 5-yr mortality after hemodialysis the calibration curve.

**Figure 7.**
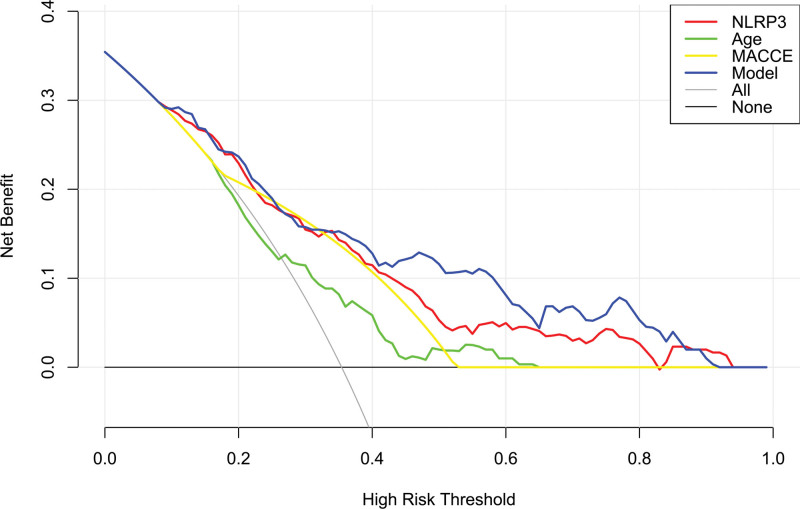
Decision curve exhibiting the clinical benefit of the prediction model of 5-yr mortality of patients with hemodialysis. The prediction model, which contained serum nucleotide-binding oligomerization domain-like receptor family pyrin domain-containing 3 levels, age and major adverse cardiac and cerebral events, was clinically beneficial in predicting the risk of 5-yr death in hemodialysis patients. MACCE = major adverse cardiac and cerebral events, NLRP3 = nucleotide-binding oligomerization domain-like receptor family pyrin domain-containing 3.

**Figure 8.**
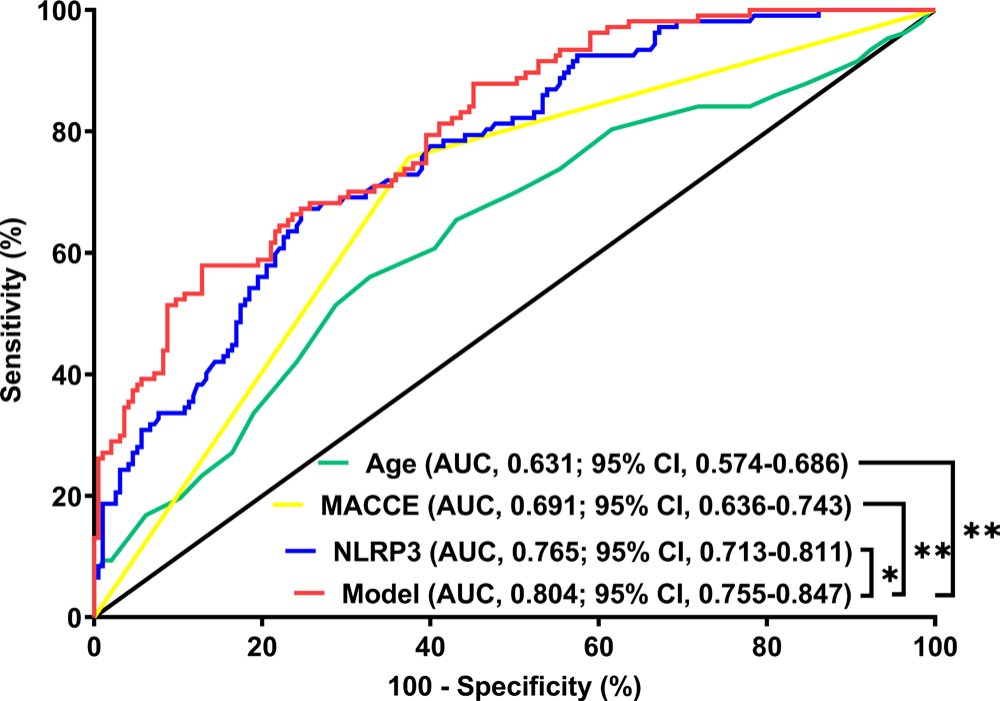
Receiver operating characteristic curve with respect to discriminatory ability of the prediction model for the risk of death in hemodialysis patients. The prediction model, which contained serum nucleotide-binding oligomerization domain-like receptor family pyrin domain-containing 3 levels, age, and major adverse cardiac and cerebral events, had a significantly higher predictive ability for the risk of death in hemodialysis patients, in contrast to each one of the preceding 3 factors (all *P* < .05). 95% CI = 95% confidence interval, AUC = area under curve, MACCE = major adverse cardiac and cerebral events, NLRP3 = nucleotide-binding oligomerization domain-like receptor family pyrin domain-containing 3. *P < .05; **P < .01.

Altogether, a 5-year follow-up was completed in 302 patients. Among them, 107 patients (35.4%) died and 195 patients (64.6%) survived. In Table 2, non-survivors were significantly older than survivors (*P* < .001), serum NLRP3 levels, serum C-reactive protein levels and blood white blood cell counts were substantially higher in the dead than in the alive (all *P* < .05), and the deceased exhibited markedly higher percentages of MACCE, hypertension, and diabetes than other remainders (all *P* < .05). The above-mentioned 7 significant variables were all forced into the binary logistic regression model and thereafter it was revealed that age, serum NLRP3 levels, and MACCE emerged as the 3 independent predictors of death in hemodialysis patients (all *P* < .05; Table 3). In Figure 2, serum NLRP3 levels were linearly related to the risk of death in hemodialysis patients under restricted cubic spline (*P* = .100). Serum NLRP3 levels significantly discriminated the risk of death in hemodialysis patients (AUC, 0.765; 95% CI, 0.713–0.811). Using the Youden method, serum NLRP3 levels more than 2.14 ng/mL predicted 5-year death with the maximum Youden index of 0.427 (Fig. 3). Interestingly, in contrast to age (AUC, 0.631; 95% CI, 0.574–0.686) and MACCE (AUC, 0.691; 95% CI, 0.636–0.743), serum NLRP3 levels had a substantially higher predictive value (*P* = .0016 and .0346, respectively). Subgroup analysis showed that no substantial interactions occurred between serum NLRP3 levels and other variables, such as age, gender, hypertension, diabetes mellitus, primary renal diseases, and MACCE (all *P* > .05; Fig. 4). In addition, the combined model was composed of serum NLRP3 level, age, and MACCE. Next, the combined model was visually reflected by a nomogram, which was constructed to assess the risk of death in hemodialysis patients (Fig. 5). The prediction model had comparative stability using calibration curve analysis (Fig. 6). Furthermore, the prediction model was clinically effective using decision curve analysis (Fig. 7) and had a significant discriminative effect on the risk of death in hemodialysis patients (AUC, 0.804; 95% CI, 0.755–0.847; Fig. 8). Moreover, the combined model had dramatically higher AUC than each one of the preceding 3 factors (all *P* < .05; Fig. 8).

**Table 2:** Differences of basic data between non-survivors and survivors among 302 hemodialysis patients.

Variables	The deceased	The alive	*P* values
Age (yr)	71.6 ± 7.4	68.5 ± 6.5	<.001
Gender (male/female)	39/68	69/126	.854
Hypertension	69 (64.5%)	101 (51.8%)	.033
Diabetes mellitus	76 (71.0%)	109 (55.9%)	.010
Primary renal diseases			.423
Chronic glomerulonephritis	49 (45.8%)	80 (41.0%)	
Others	58 (54.2%)	115 (59.0%)	
Major adverse cardiac and cerebral events	81 (75.7%)	73 (37.4%)	<.001
Laboratory data Blood intact parathyroid hormone levels (pg/mL)	400.7 (249.9–705.4)	342.3 (120.8–688.5)	.072
Blood creatinine levels (mg/dL)	9.4 (8.5–11.2)	9.2 (7.9–10.8)	.143
Blood albumin levels (mg/dL)	3.5 ± 0.8	3.4 ± 0.8	.392
Blood hemoglobin levels (g/dL)	10.5 ± 2.5	10.2 ± 2.4	.299
Hematocrit (%)	29.1 (25.5–34.2)	27.9 (23.8–33.2)	.263
Blood alkaline phosphatase levels (IU/L)	89.4 ± 19.7	85.6 ± 20.6	.124
Blood C-reactive protein levels (mg/dL)	1.1 (0.8–1.5)	1.0 (0.5–1.4)	.010
Blood cholesterol levels (mg/dL)	182.2 (162.3–212.6)	174.6 (149.9–207.8)	.219
Blood triglyceride levels (mg/dL)	121.2 (83.3–168.0)	106.3 (64.3–164.2)	.315
Blood leucocyte count (×10^9^/L)	7.6 ± 2.0	7.0 ± 1.8	.015
Blood calcium levels (mg/dL)	9.1 (8.2–10.8)	8.8 (7.5–10.4)	.143
Blood phosphate levels (mg/dL)	5.0 (4.3–5.9)	4.7 (4.1–5.6)	.147
Blood calcium × phosphate levels	44.6 (40.1–53.3)	44.2 (37.0–52.6)	.436
Urea reduction rate (%)	0.8 ± 0.2	0.7 ± 0.2	.060
Post-dialysis weight (kg)	53.6 (48.2–63.3)	52.4 (44.7–62.4)	.293
Kt/V (Daugirdas)	1.7 ± 0.4	1.6 ± 0.4	.068
Normalized protein catabolic rate (g/kg/d)	1.2 ± 0.2	1.1 ± 0.3	.089
Residual creatinine clearance (mL/min)	5.5 (4.9–6.5)	5.2 (4.4–6.2)	.057
Serum NLRP3 levels (ng/mL)	2.8 (1.9–3.7)	1.6 (0.9–2.1)	<.001

Data were shown as counts (percentages), means ± standard deviations, or medians (upper-lower quartiles) where appropriate. Intergroup comparisons were done using the Chi-square test, Fisher exact test, Student *t* test, or Mann–Whitney test as appropriate.

NLRP3 = nucleotide-binding oligomerization domain-like receptor family pyrin domain-containing 3.

**Table 3:** Multivariate logistic regression analysis of death among 302 hemodialysis patients.

Variables	Odds ratio	95% confidence interval	*P* value
Age (yr)	1.043	1.000–1.088	.047
Hypertension	1.009	0.545–1.865	.978
Diabetes mellitus	1.581	0.848–2.948	.149
Major adverse cardiac and cerebral events	2.383	1.283–4.426	.006
Blood C-reactive protein levels (mg/dl)	1.529	0.915–2.556	.105
Blood leucocyte count (×10^9^/L)	1.042	0.884–1.230	.623
Serum NLRP3 levels (ng/mL)	2.149	1.634–2.827	.001

NLRP3 = nucleotide-binding oligomerization domain-like receptor family pyrin domain-containing 3.

### 3.3. Serum NLRP3 levels and 5-year overall survival of hemodialysis patients

**Figure 9.**
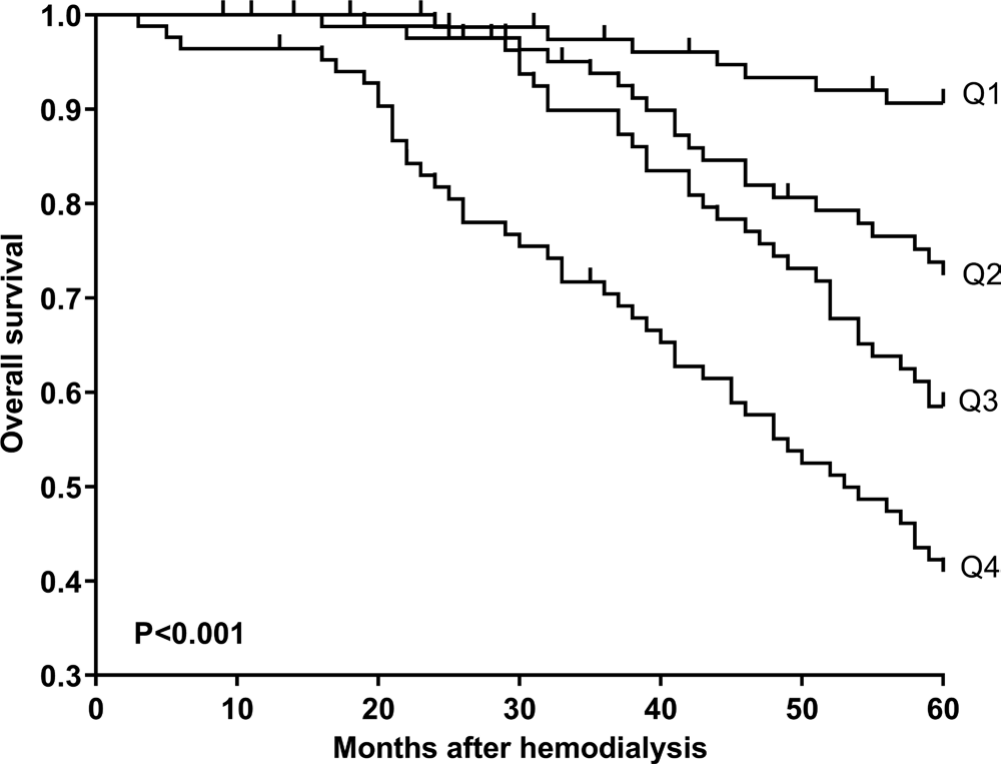
Survival curve showing 5-yr overall survival after hemodialysis across serum nucleotide-binding oligomerization domain-like receptor family pyrin domain-containing 3 levels. Patients were divided into 4 groups according to percentiles 25th, 50th, and 75th of serum nucleotide-binding oligomerization domain-like receptor family pyrin domain-containing 3 levels: Q1, Q2, Q3, and Q4. Using the log-rank test, survival rates decreased significantly with an increase in serum nucleotide-binding oligomerization domain-like receptor family pyrin domain-containing 3 levels (*P* < .001).

**Figure 10.**
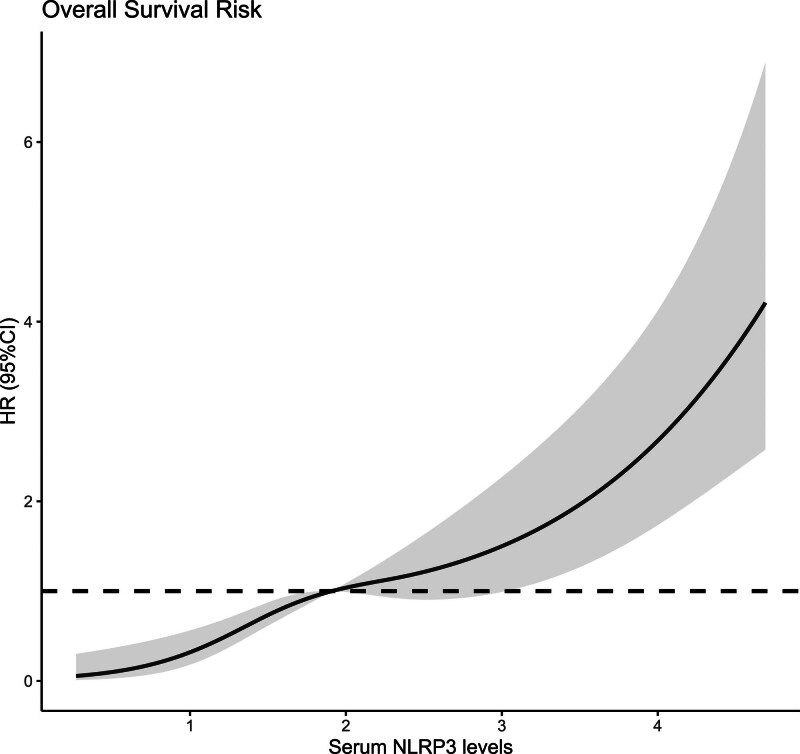
Restricted cubic spline ascertaining the linear relation of serum nucleotide-binding oligomerization domain-like receptor family pyrin domain-containing 3 levels to the risk of 5-yr overall survival after hemodialysis. Serum nucleotide-binding oligomerization domain-like receptor family pyrin domain-containing 3 levels were linearly correlated with the risk of 5-yr overall survival in hemodialysis patients (*P* > .05). 95% CI = 95% confidence interval, HR = hazard ratio, NLRP3 = nucleotide-binding oligomerization domain-like receptor family pyrin domain-containing 3.

**Figure 11.**
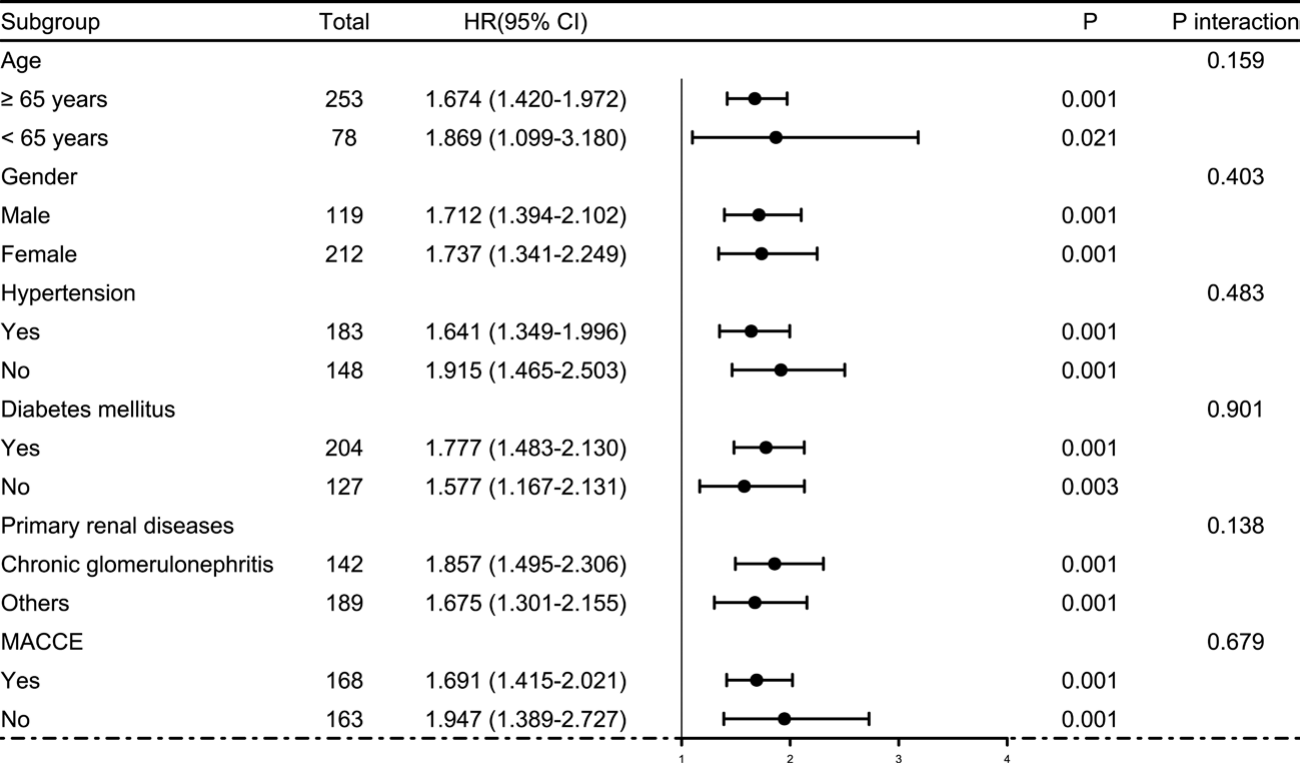
Subgroup analysis assessing interaction between serum nucleotide-binding oligomerization domain-like receptor family pyrin domain-containing 3 levels and other variables for predicting 5-yr overall survival of hemodialysis patients. Serum nucleotide-binding oligomerization domain-like receptor family pyrin domain-containing 3 levels had no substantial interaction with age, gender, hypertension, diabetes mellitus, primary renal diseases, and major adverse cardiac and cerebral events (all *P* > .05). 95% CI = 95% confidence interval, HR = hazard ratio, MACCE = major adverse cardiac and cerebral events.

**Figure 12.**
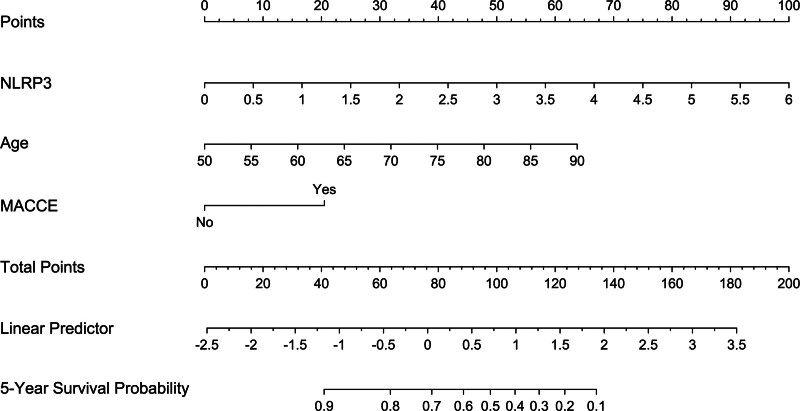
Nomogram describing the prediction model of the probability of 5-yr overall survival of hemodialysis patients. A nomogram, in which serum nucleotide-binding oligomerization domain-like receptor family pyrin domain-containing 3 levels, age, and major adverse cardiac and cerebral events were merged, was constructed to visually display the prediction model of 5-yr overall survival of patients with hemodialysis. Draw a line down to the morbidity axes to determine the possibility of 5-yr overall survival of hemodialysis patients. MACCE = major adverse cardiac and cerebral events, NLRP3 = nucleotide-binding oligomerization domain-like receptor family pyrin domain-containing 3.

**Figure 13.**
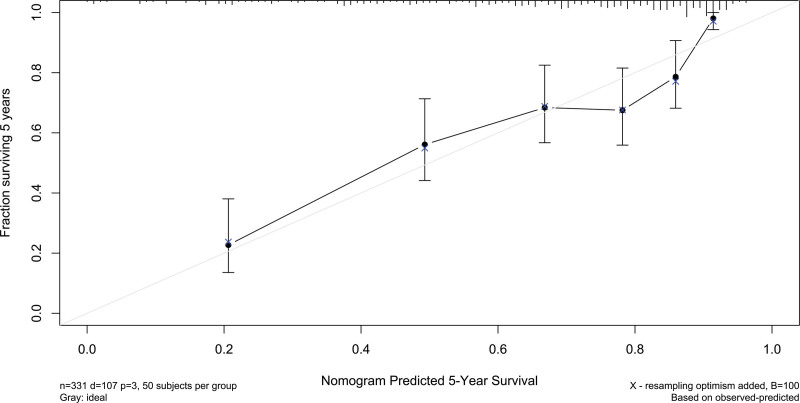
Calibration curve exhibiting the stability of the prediction model of 5-yr overall survival of patients with hemodialysis. The prediction model, which contained serum nucleotide-binding oligomerization domain-like receptor family pyrin domain-containing 3 levels, age and major adverse cardiac and cerebral events, was stable in predicting 5-yr overall survival in hemodialysis patients.

**Figure 14.**
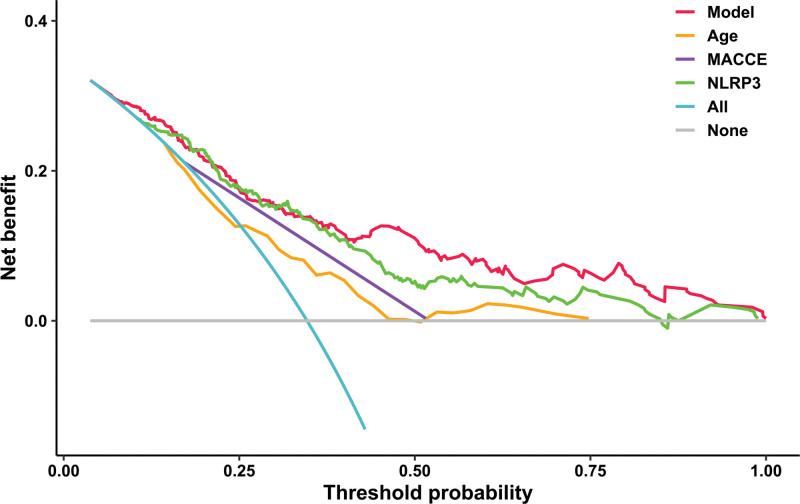
Decision curve exhibiting the clinical benefit of the prediction model of 5-yr overall survival of patients after hemodialysis. The prediction model, which contained serum nucleotide-binding oligomerization domain-like receptor family pyrin domain-containing 3 levels, age and major adverse cardiac and cerebral events, was clinically beneficial in predicting 5-yr overall survival in hemodialysis patients. MACCE = major adverse cardiac and cerebral events, NLRP3 = nucleotide-binding oligomerization domain-like receptor family pyrin domain-containing 3.

Mean 5-year overall survival time was 53.0 months (95% CI, 51.6–54.4) in 331 patients. Patients were divided into 4 groups according to percentiles 25th, 50th, and 75th of serum NLRP3 levels: Q1 (0.21–1.13), Q2 (1.14–1.90), Q3 (1.91–2.93), and Q4 (2.94–5.96). The 5-year death rates in the 4 groups were 8.6%, 25.3%, 38.6%, and 56.0%, respectively. Using the Log-rank test, survival rates decreased significantly with an increase in serum NLRP3 levels (*P* < .001; Fig. 9). In Figure 10, serum NLRP3 levels were linearly correlated with overall survival risk under restricted cubic spline (*P* = .098). Table 4 shows that age, diabetes mellitus, hypertension, MACCE, serum NLRP3 levels, serum C-reactive protein levels, and blood white blood cell counts were significantly associated with 5-year overall survival of hemodialysis patients (all *P* < .05). The above-mentioned 7 significant variables were all forced into the multivariate Cox regression model and afterward it was found that serum NLRP3 levels, age and MACCE retained as the 3 independent predictors of 5-year overall survival in hemodialysis patients (all *P* < .05; Table 5). Also, there were no marked interactions between serum NLRP3 levels and age, gender, hypertension, diabetes mellitus, primary renal diseases, and MACCE (all *P* > .05; Fig. 11). The nomogram was constructed to predict the probability of 5-year overall survival of hemodialysis patients (Fig. 12). The calibration curve showed good agreement between predicted and observed outcomes for 5-year overall survival prediction model (Fig. 13). Decision curve analysis confirmed the clinical effectiveness of the prediction model (Fig. 14).

**Table 4:** Factors in relation to overall survival among 331 hemodialysis patients.

Variables	Hazard ratio	95% confidence interval	*P* value
Gender (male/female)	0.954	0.643–1.414	.813
Age (yr)	1.068	1.036–1.101	<.001
Hypertension	1.518	1.022–2.256	.039
Diabetes mellitus	1.713	1.128–2.601	.012
Primary renal diseases			
Chronic glomerulonephritis	1		
Others	0.861	0.588–1.259	.440
Major adverse cardiac and cerebral events	3.839	2.465–5.976	<.001
Laboratory data			
Blood intact parathyroid hormone levels (pg/mL)	1.000	1.000–1.001	.278
Blood creatinine levels (mg/dL)	1.055	0.967–1.150	.227
Blood albumin levels (mg/dL)	1.082	0.859–1.363	.502
Blood hemoglobin levels (g/dL)	1.032	0.956–1.114	.421
Hematocrit (%)	1.012	0.985–1.040	.380
Blood alkaline phosphatase levels (IU/L)	1.006	0.997–1.051	.197
Blood C-reactive protein levels (mg/dL)	1.418	1.062–1.891	.018
Blood cholesterol levels (mg/dL)	1.002	0.998–1.007	.328
Blood triglyceride levels (mg/dL)	1.001	0.998–1.004	.487
Blood leucocyte count (×10^9^/L)	1.126	1.017–1.246	.022
Blood calcium levels (mg/dL)	1.055	0.967–1.151	.229
Blood phosphate levels (mg/dL)	1.107	0.943–1.299	.213
Blood calcium × phosphate levels	1.005	0.987–1.022	.602
Urea reduction rate (%)	2.463	0.939–6.461	.067
Post-dialysis weight (kg)	1.006	0.991–1.021	.440
Kt/V (Daugirdas)	1.628	0.969–2.736	.066
Normalized protein catabolic rate (g/kg/d)	1.653	0.816–3.349	.163
Residual creatinine clearance (mL/min)	1.151	0.993–1.333	.062
Serum NLRP3 levels (ng/mL)	1.895	1.642–2.188	<.001

Univariate Cox proportional hazard regression analysis was performed.

NLRP3 = nucleotide-binding oligomerization domain-like receptor family pyrin domain-containing 3.

**Table 5:** Multivariate Cox proportional hazard regression analysis of overall survival among 331 hemodialysis patients.

Variables	Hazard ratio	95% confidence interval	*P* value
Age (yr)	1.050	1.017–1.084	.003
Hypertension	0.946	0.623–1.436	.793
Diabetes mellitus	1.349	0.867–2.100	.185
Major adverse cardiac and cerebral events	1.937	1.188–3.159	.008
Blood C-reactive protein levels (mg/dL)	1.312	0.933–1.846	.199
Blood leucocyte count (×10^9^/L)	0.986	0.873–1.114	.821
Serum NLRP3 levels (ng/mL)	1.720	1.472–2.009	.001

NLRP3 = nucleotide-binding oligomerization domain-like receptor family pyrin domain-containing 3.

## 4. Discussion

To the best of our knowledge, it is unclear whether serum NLRP3 levels may be correlated with 5-year mortality in incident hemodialysis patients. In this study, we found that serum NLRP3 levels were significantly increased with decreasing 5-year overall survival time and were substantially higher in the deceased than in the alive within 5 years after hemodialysis; serum NLRP3 levels were independently associated with 5-year mortality and overall survival of patients with hemodialysis; 2 models containing serum NLRP3 levels, age, and MACCE performed well in prediction of death and overall survival at 5 years after hemodialysis in such patients. Overall, the preceding data are strongly supportive of the presumption that serum NLRP3 may be of clinical significance as a potential prognostic biomarker in hemodialysis patients.

Among patients undergoing hemodialysis, MACCE is easily encountered, which is associated with significantly heightened risk of death.^[24,25]^ Reportedly, MACCE incidence within a 2-year time frame ranges from 10.3% to 36.7% for those patients.^[26–29]^ In this cohort, 5-year MACCE incidence was approximately 50%, indicating that its incidence may be significantly elevated with increasing follow-up time. MACCE has been demonstrated to be an independent risk factor of death in hemodialysis.^[30]^ Consistently, MACCE was independently associated with 5-year mortality in our study. Taken together, MACCE may be a common complication of patients under hemodialysis, which should be paid intensive attention and treated during hemodialysis.

ESRD is very frequently complicated with other tissue injuries.^[31]^ Hemodialysis patients mainly die of multiple organ dysfunction or failure, which is related to systemic injures.^[32]^ Pathophysiological mechanisms underlying systemic injures in ESRD involve inflammation, oxidative stress, cellular apoptosis, etc.^[33]^ Thus, a circulating biomarker, which is able to reflect status of systemic injuries, may be a potential predictor of death among hemodialysis patients.

NLRP3 participates in inflammatory processes of several severe illnesses, such as acute myocardial infarction, stroke, sepsis, and pneumonia.^[17–19,34,35]^ Alternatively, elevated circulating NLRP3 levels were highly related to disease severity and poor prognosis of those diseases.^[17–19,34]^ Moreover, renal mononuclear phagocytes, such as dendrites and macrophages, could express NLRP3, which was involved in the acute and chronic inflammation of the kidneys by inducing the secretion of interleukin-1β and interleukin-18.^[36–41]^ Diabetic and hypertensive nephropathies are the 2 main causes of ESRD. In a study of diabetic nephropathy, which contained in vitro and in vivo data, NLRP3 inflammasome was found to be activated.^[42]^ NLRP3 inflammasome activity may be involve in the blood pressure fluctuation and kidney injury.^[43]^ Overall, NLRP3 may be a potential systemic inflammatory biomarker, which could reflect not only renal injury, but also systemic injury.

In this study, a total of 331 finally recruited patients were included in compliance with inclusion and exclusion requirements. To investigate the association between serum NLRP3 levels and 5-year overall survival of hemodialysis patients, patients were divided into 4 subgroups based on the tertiles of serum NLRP3 levels. Clearly, the 5-year overall survival rates were significantly declined with increasing serum NLRP3 levels from Q1 to Q4 among patients. Moreover, using multivariate analysis, serum NLRP3 levels were independently associated with 5-year overall survival of patients undergoing hemodialysis. In addition, a total of 302 were followed up until death or the completion of 5 years, and subsequently were divided into 2 subgroups, namely, the deceased and the alive groups. As opposed to all patients, this group of patients had non-statistically significant differences in terms of demographic, clinical, and biochemical data, indicating this group of patients could represent the whole group of patients. Consistently, serum NLRP3 levels were independently associated with 5-year death after hemodialysis. The above data were strongly supportive of the assumption that higher serum NLRP3 levels may be linked to higher risk of 5-year mortality in hemodialysis patients. Thus, serum NLRP3 may be a potential biomarker of long-term death among hemodialysis patients.

In present study, serum NLRP3 levels were linearly correlated with death of hemodialysis patients. Besides serum NLRP3, the other 2 independent factors of 5-year death were age and MACCE, which have been confirmed to be commonest predictors of death among hemodialysis patients. In current study, serum NLRP3, age, and MACCE had AUCs at 0.765, 0.631, and 0.691, respectively. Among them, serum NLRP3 displayed significantly highest death prediction ability in terms of AUC. Moreover, serum NLRP3, age, and MACCE were merged in a prediction model, which was revealed to take possession of significantly highest AUC, as compared to other 3 predictors alone. The model was visually described using a nomogram. Using calibration curve analysis and decision curve analysis, the model was comparatively stable and clinically valuable for predicting 5-year death of hemodialysis patients. This indicates that the combined model can be used as a good predictor of mortality risk in hemodialysis patients.

There are several strengths and weaknesses in the current study. The strengths are that to the best of our knowledge, serum NLRP3 levels were determined in hemodialysis patients for the first time, and subsequently it was found that serum NLRP3 may be a useful prognostic biomarker of hemodialysis patients with ESRD; in order to offer more evidence to support the conclusion, both 5-year mortality and overall survival were applied to reflect prognosis of hemodialysis patients, and associations of serum NLRP3 levels with them were verified using univariate analysis followed by multivariate analysis. The weaknesses are that the present study was based on Chinese patients. Further validation with diverse populations and races is essential; the present study is a single-center study and therefore the reproducibility of the current findings should be further validated in a larger cohort study or a multicenter study.

## 5. Conclusions

Serum NLRP3 levels of hemodialysis patients are independently associated with 5-year mortality and overall survival. Under ROC curve, serum NLRP3 levels show higher death prediction ability, as compared to other 2 independent predictors, that is, age and MACCE. Meanwhile, the combined model, which is composed of serum NLRP3 levels, age, and MACCE, significantly improves their discriminatory efficiency for the risk of death in hemodialysis patients. Moreover, such a model is of stability and has comparatively high clinical value. Thus, serum NLRP3 may be used as a good predictor of 5-year mortality of patients undergoing hemodialysis.

## Acknowledgments

We gratefully thank all study participants, their relatives, and the staff at the recruitment centers for their invaluable contributions.

## Author contributions

**Conceptualization:** Yi Jiang, Zhiwei Chen.

**Investigation:** Yi Jiang, Yandan Xu, Qiuli Wang, Zhiwei Chen, Chunya Liu.

**Methodology:** Yi Jiang, Chunya Liu.

**Writing—original draft:** Yi Jiang, Yandan Xu, Zhiwei Chen, Chunya Liu.

**Writing—review & editing:** Yi Jiang, Zhiwei Chen, Chunya Liu.

**Software:** Yandan Xu, Qiuli Wang, Zhiwei Chen.

**Data curation:** Qiuli Wang.

**Formal analysis:** Qiuli Wang.

**Validation:** Qiuli Wang, Chunya Liu.
